# Molecular characterization of hepatitis B virus in Vietnam

**DOI:** 10.1186/s12879-017-2697-x

**Published:** 2017-08-31

**Authors:** Thi Ton Taht Bui, Tan Thanh Tran, My Ngoc Nghiem, Pierre Rahman, Thi Thanh Thanh Tran, Man Nguyen Huy Dinh, Manh Hung Le, Van Vinh Chau Nguyen, Guy Thwaites, Motiur Rahman

**Affiliations:** 1 Hospital for Tropical Diseases, Ho Chi Minh City, Vietnam; 2 Oxford University Clinical Research Unit, Toronto, Canada; 3 Mount Sinai Hospital, Toronto, Canada; 4 The Hospital for Tropical Diseases, Wellcome Trust Major Overseas Programme and Centre for Tropical Medicine, Nuffield Department of Clinical Medicine, Oxford University, Oxford, UK; 5 Laboratories, Centre for Tropical Medicine, Oxford University Clinical Research Unit, 764 Vo Van Kiet Street, Ward 1, District 5, Ho Chi Minh City, Vietnam

**Keywords:** Hepatitis B, Vietnam, Genotype, Subgenotype

## Abstract

**Background:**

Hepatitis B virus (HBV) infection is a major public health problem globally. HBV genotypes and subgenotypes influence disease transmission, progression, and treatment outcome. A study was conducted among treatment naive chronic HBV patients in southern Vietnam to determine the genotypes and subgenotypes of HBV.

**Methods:**

A prospective, exploratory study was conducted among treatment naïve chronic HBV patients attending at the Hospital for Tropical Diseases, in Ho Chi Minh City, Vietnam during 2012, 2014 and 2016. HBV DNA positive samples (systematically selected 2% of all treatment naïve chronic patients during 2012 and 2014, and 8% of all treatment naïve chronic patients during 2016) were subjected to whole genome sequencing (WGS) either by Sanger or Illumina sequencing. WGS was used to define genotype, sub-genotype, recombination, and the prevalence of drug resistance and virulence-associated mutations.

**Results:**

One hundred thirty five treatment naïve chronic HBV patients including 18 from 2012, 24 from 2014, and 93 from 2016 were enrolled. Of 135 sequenced viruses, 72.6% and 27.4% were genotypes B and C respectively. Among genotype B isolates, 87.8% and 12.2% were subgenotypes B4 and B2 respectively. A G1896A mutation in the precore gene was present in 30.6% of genotype B isolates. The genotype C isolates were all subgenotype C1 and 78.4% (29/37) of them had at least one basal core promoter (BCP) mutation. A1762T and G1764 T mutations and a double mutation (A1762T and G1764 T) in the BCP region were significantly more frequent in genotype C1 isolates (*p* < 0.001).

**Conclusion:**

HBV genotype B including subgenotype B4 is predominant in southern Vietnam. However, one fourth of the chronic HBV infections were caused by subgenotype C1.

**Electronic supplementary material:**

The online version of this article (10.1186/s12879-017-2697-x) contains supplementary material, which is available to authorized users.

## Background

Worldwide, an estimated 2 billion people have been infected with the hepatitis B virus (HBV) and of these 250 million suffer from chronic HBV infection [1].HBV infection either leads to spontaneous recovery or to chronic HBV, which causes chronic liver disease, including liver cirrhosis (LC) and hepatocellular carcinoma (HCC) [2].

HBV is a small circular DNA virus (~3.2 kb in length) that contains 4 genes with partially overlapping open reading frames (ORFs). These overlapping ORFs encode the polymerase protein, the surface antigen, the core antigen and the X protein. HBV is highly heterogeneous and is composed of genomes that are closely related but not identical; hence, it is considered as a viral quasispecies within an infected individual [3]. Viral replication is rapid with up to 10^11^ virions generated each day in infected individual.Due to the high reverse transcription error rate of the polymerase (1 error/10^7^ bases) during active infection, 10^7^ base-pairing errors can be generated over the 3200-bp genome per day [4].While most of these new sequences are within nonviable viruses, they provide a starting point for the emergence of mutants under selective pressure.

HBV can be categorized into 10 different genotypes (A-J; segregated by <7.5% genomic sequence diversity) and 40 different subgenotypes (separated by >4% genomic sequence diversity) [5]. HBV subgenotyping has caused controversy in the past due to misclassifications and incorrect interpretations from different genotyping methods (whole genome sequence versus S gene sequence). Criteria for assigning a new subgenotype have been proposed recently [5]. They include: i) analysis of full-length genome, ii) adherence to intra-genotypic nucleotide divergence (>4.5% and <7.5%), iii) bootstrap values greater than 75%, iv) exclusion of recombinant strains from analysis, v) identification of specific nucleotide and amino acid motifs, vi) a minimum of three purported novel strains, and vii) all available subgenotype strains belonging to the same genotype should be subjected to evolutionary and phylogeny analysis.

HBV genotypes and sub-genotypes differ considerably with respect to geographical distribution, transmission routes, disease progression, responses to antiviral therapy, and clinical outcome, e.g. LC and HCC [6]. The clinical course of infection depends on the host’s age at infection, genetic factors, and the genomic variability of the virus, including genotypes, subgenotypes and virulence associated mutations [*preS*1, *preS*2, *S* gene mutations, basal core promoter (BCP), precore (PC) and core mutations [7–9]. Earlier studies have shown that the mutations in the *preS*1 and *preS*2 genes are associated with progression to HCC [9]. The AA positions between 99 to 169 in the S gene are called the major hydrophobic region (MHR) and the “a” determinant region (aa 124 to 147) is located within the MHR. Mutations in the “a” determinant region cause conformational changes in the S protein that can affect the antigenicity of HBsAg and can generate immune escape mutants [10]. Mutations in BCP, PC and core have shown to be associated with HCC [11].

Vietnam is classified as a high burden country regarding hepatitis, and the prevalence of chronic HBV infection is 8–20% and 31–54% among the general and the urban high risk populations respectively [12]. Projection and modeling studies have predicted approximately 8 million chronic HBV cases and 58,600 HBV related LC cases in Vietnam by 2025, and that the estimated annual HBV related mortality will be 40,000/year by 2025 [13]. Limited research has been conducted on molecular characterization and determination of virulence associated properties of HBV in Vietnam. Representative data on genotype and subgenotype analysis using whole genome sequence (WGS) [14–16], as well as information on the prevalence of virulence associated mutations in different genes, recombination and drug resistant mutations in treatment naïve patients are limited.

We conducted a prospective exploratory study using systematically and randomly collected HBV isolates from treatment naïve patients attending a tertiary care hospital in southern Vietnam from 2012, 2014 and 2016 respectively. We used WGS to investigate the prevalences of i) genotype and subgenotype, ii) recombinants, iii) primary, secondary and potential nucleos(t)ide analogue (NA) resistance (NAr) mutations, and iv) mutations in the *preS*1, *preS*2, S gene, BCP/PC and core gene.

## Methods

The study was conducted at the Hospital for Tropical diseases (HTD), Ho Chi Minh City Vietnam from June to December 2012, January to June 2014 and January to December 2016. HTD is a 650 bed tertiary care hospital for infectious diseases and a designated referral center for hepatitis patients for the southern provinces of Vietnam. All treatment naïve chronic HBV patients attending at the hepatitis outpatient department for viral load assays were eligible for the study. Systematically selected residual diagnostic samples from 2% of the patients (samples from every 50th patient) from 2012 and 2014 and 8% of the patients from 2016 were included in this study. Serum samples from selected patients were stored at minus 86 °C until further analysis. Patient address (province, district, city, and wards), clinical chemistry and viral load data were collected from the hospital database. The geolocation of the patients were mapped with QGIS software version 2.18. The study was approved by the Hospital for Tropical Diseases’ ethical review committee (Approval No: SC/ND/12/14).

Viral DNA was extracted from 200 μL of plasma using QIAamp viral DNA kit (QIAgen GmbH, Hilden, Germany) and eluted in 50 μL TBE. The HBV genome was amplified in 4 overlapping fragments (800 bp to 1.2 kb) using P1-P2, P3-P4, P5-P6, and P7-P8 primers (P1: 5′-TTT TTC ACC TCT GCC TAA TCA-3′; P2: 5′-TTG GGA TTG AAG TCC CAA TCT GG-3′; P3: 5′-GGG TCA CCT TAT TCT TGG-3′; P4: 5′-ATA ACT GAA AGC CAA ACA GTG GG-3′; P5: 5′-GTC TTC TTG GTT GTT CTT CTA C-3′; P6: 5′-GCA GCA CAG CCT AGC AGC CAT GG-3′; P7: 5′-CCA TAC TGC GGA ACT CCT AGC-3′; P8: 5′-CAA TGC TCA GGA GAC TCT AAG GC-3′) [17]. PCR reaction was done in 40 μL of buffer containing 50 mM Tris-HCl (pH 8.3), 50 mM KCl, 1.5 mM MgCl2, 200 mM deoxynucleoside triphosphates (dNTPs), 1 U of Taq DNA-Pwo Polymerase (Expand High Fidelity assay, Boehringer Mannheim), and 30 pmol of primers. The PCR was performed for 35 cycles at 94 °C for 1 min, 58 °C for 1 min, and 72 °C for 1 min in a thermal cycler (ABI 9800) [18]. The PCR products were visualized by 1% agarose electrophoresis and stained with Nancy 520 DNA gel stain. The PCR product was purified using QIAamp PCR product purification kit (QIAgen GmbH, Hilden, Germany). The eluted DNA was quantified by a fluorescence-based dsDNA quantification method using the Quant-iT dsDNA Assay Kit in a Qubit fluorometer (Invitrogen) and was sequenced either by ABI 3100 system after cycle sequencing reaction or by Illumina Myseq system. For the ABI 3100 system, DNA sequencing was done from both ends and consensus sequence was used to construct the whole genome using overlapping fragments. For Illumina sequencing, the amplified fragments were pooled with an equal quantity of each individual PCR amplicon. One nanogram of pooled DNA from individual samples was subjected to library preparation using the Nextera XT DNA sample preparation kit (Illumina, San Diego, CA, USA), in which each sample was assigned to a unique barcode sequence using the Nex-tera XT Index Kit (Illumina). Sequencing of the prepared library was carried out using the Miseq reagent kit v2 (300 cycles, Illumina) in an Illumina Miseq platform.

The Illumina fastq sequence files were assembled using Genious 8.0.5, software package (Biomatters Ltd, AK, New Zeland) utilizing a reference-based mapping tool after primer sequence clipping (i.e. the consensus sequence was obtained by mapping individual reads of each sample to a reference sequence). Finally, screening of minor (sub-consensus) variants was performed using the SNP detection tool available in Geneious. A minimum variant frequency of 5% and 500-fold coverage were chosen as cut-off values.

Seventy well characterized HBV WGS representing all genotypes and subgenotypes were downloaded from Gene Bank and the HBV WGS from the current study were subjected to phylogenetic analysis. All complete genome sequences were aligned with MUSCLE from the Genious package. The sequence alignments were then subjected to the Jmodel test to identify the best model for phylogenetic analysis [19]. The suggested nucleotide substitution model (GTR + G + I) was subsequently used in phylogenetic analysis using RAxML v7.2.8 (available in the Genious package). To confirm the reliability of phylogenetic tree analysis, bootstrap resampling and reconstruction were carried out 100 times.

All sequences were analyzed for possible recombination by RDP4 v 4.85 software [20]. Any recombination events detected by at least 5 of the 7 programs (RDP, Geneconv, Bootscan, Maxchi, Chimaera, Siscan and Topol) were considered as true recombination. RDP4 v4.85 standard default settings were used, except for Bootscan and Siscan where window sizes of 300 bp, step size 30 were used. The prevalence of recombination, recombination breakpoints (start and end point), length of the recombinant fragments and the locations of the recombination were determined.

HBV reverse transcriptase (RT) regions were analyzed for the presence or absence of 42 potential nucleos(t)ide analogue (NA) resistance (NAr) mutations. This includes primary and secondary drug resistance mutations (rt80, rt169, rt173, rt180, rt181, rt184, rt194, rt202, rt204, rt236 and rt250), putative NAar mutations (rt53, rt54, rt82, rt84,rt85, rt91, rt126, rt128, rt139, rt153,rt166, rt191,rt200, rt207,rt213, rt214,rt215,rt217, rt218, rt221, rt229, rt233, rt237, rt238, rt245, and rt256), and pretreatment mutations (rt38, rt124, rt134, rt139, rt224 and rt242) as described earlier [21].

The *preS2/S1* sequences were analyzed for *preS1* deletion, *preS1* mutations (A2962G, C3026A/T, C2964A, and C3116T), *preS2* start codon deletion, and pre*S2* mutations (T31C, T53C, A162G, and T531C/G). The S gene sequence was analyzed for mutations in the “a” determinant region (T116 N, P120S/T, I/T126S/A, Q129H/R, M133 L/T, K141E, P142S, D144E, and G145R), and other virulence associated mutations (N3S, V184A, and S204R).

Mutations in the BCP (C1653T, T1674C/G, T1753 V, A1762T, G1764/A, C1766T, and T1768A) and the PC/core region (G1899A, C2002T, A2159G, A2189C, and G2203A/T) associated with HCC were also analyzed.

All data (socio demographic, biochemical and virological) were recorded and analyzed with Statistical Package for the Social Sciences (IBM SPSS version 23, NY, USA). Fisher’s exact test was used for the comparison of nominal scale variables and Mann - Whitney U test for ordinal scale variable. A *P* value </0.05 was considered to indicate statistically significant difference.

## Results

From June to December 2012, January to June 2014, and June to December 2016 a total of 11,575, 10,329 and 18,141 chronic HBV patients submitted blood samples for HBV viral load assay, and of those 912, 1245 and 1174 patients were treatment naïve respectively. Eighteen samples from 2012 and twenty four from 2014 (2% of the systematically collected samples) and ninety three samples from 2016 (8% of the samples) were enrolled in the study.

The geolocations of the 135 patients enrolled in the study were mapped; 87 districts were represented from the 26 southern provinces of Vietnam. Approximately 58% of the patients were from six provinces including Ho Chi Minh City (28.1%; 38/135), Dong Nai (6.66%; 9/135), Long Anh (6.66%; 9/135), Binh Duong (5.93%; 8/135), Dong Thap (5.18%; 7/135) and Tien Giang (5.18%; 7/135). The rest of the patients were from 46 districts of the remaining 20 southern provinces of Vietnam (Additional file 1). The gender, demographic information, liver enzyme level, viral load and genotype and subgenotype distribution of 135 patients are presented in Table 1. Approximately half of the patients were male and the mean age of the patients was 32 years. 11.1% (15/135) had a blood ALT concentration of ≥5UNL (reference range is <37 IU/l and <40 IU/l, for females and males, respectively).

**Table 1:** Socio-demographic, biochemical and virological characteristics of 135 patients enrolled in 2102, 2014 and 2016

Parameter	All	Genotype B	Genotype C	*p* value
	% (n)	% (n)	% (n)	
	100 (135)	72.6 (98)	27.4 (37)	
Subgenotype				
B2	8.9 (12)	12.2 (12)		
B4	63.7 (86)	87.7 (86)		
C1	27.4 (37)		100 (37)	
Age (mean ± SD)	33.0 ± 11.6	31.9 ± 11.3	35.8 ± 12.2	0.036
Sex				
Male	51.9 (70)	53.1 (52)	48.6 (18)	0.395†
Female	48.14 (65)	46.9 (46)	51.4 (19)	
Year of enrolment				
2012	13.3 (18)	13.3 (13)	13.5 (5)	
2014	17.8 (24)	19.4 (19)	13.5 (5)	
2016	68.9 (93)	67.3 (66)	73.3 (27)	
Biochemistry (median (range)				
AST	10 (15.0–707.0)	50.5 (15.0–707.0)	53.5 (20.0–471.0)	0.509*
ALT	69.5 (9.0–1025.0)	67.5 (9.0–1025.0)	72.5 (9.0–594.0)	0.471*
Creatinine	83.0 (11.0–126.0)	83.0 (11.0–114.0)	85.0 (45.0–126.0)	0.392*
Bilirubin	13.1 (6.0–360.0)	13.2 (6.0–198.0)	12.8 (7.3–360.0)	0.838*
HBV Viral load				
Mean	1.45 × 10^9^	1.51 × 10^9^	1.29 × 10^9^	
(Min–Max)	2.03 × 10^6^–9.35 × 10^9^	2.03 × 10^6^–6.28 × 10^9^	2.65 × 10^6^–9.35 × 10^9^	
Median	8.01 × 10^8^	9.29 × 10^8^	3.05 × 10^8^	0.059*

†Fisher’s exact test; *Mann-Whitney U test

**Fig. 1:**
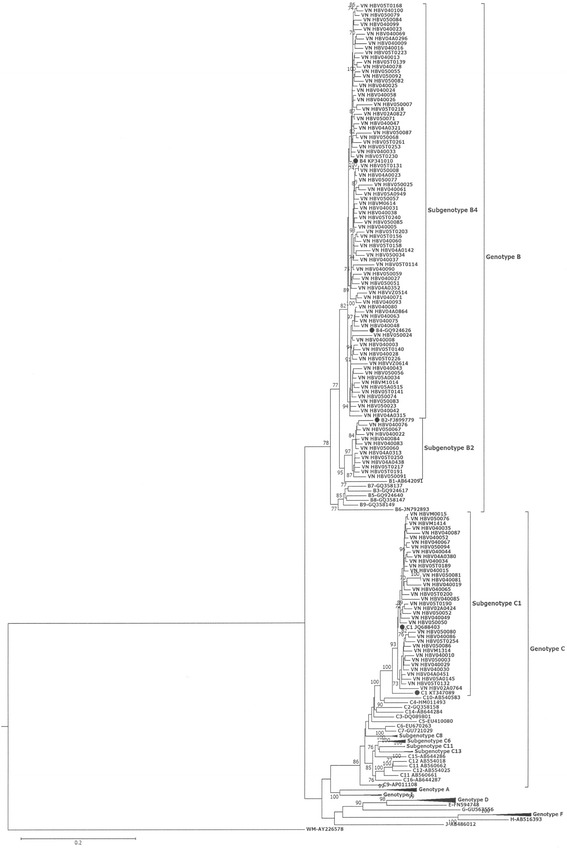
A midpoint rooted tree showing the relationship between the Vietnamese HBV genome sequences with 70 reference sequences (all genotypes and subgenotypes). Tree was constructed using RAxML v7.2.8 available in Geneious software using GTR + G + I nucleotide substitution model with 100 bootstrapping replicates. Bootstrap values greater than 70% are shown on the branch nodes. The Vietnamese HBV strains are presented as VN HBV followed by isolate number and reference genomes are presented as subgenotype followed by Gene Bank accession number. The reference genomes (B2, B4 and C1) are highlighted with a filled dark circle. Reference genomes with multiple sequences for each genotype and subgenotypes (genotype and subgenotype A, D, F, I and subgenotype C6, C8 and C13) were clustered. The scale bar indicates the number of nucleotide substitution. Genotype, subgenotype and Gene Bank accession number of reference sequences are listed in Additional file 3

The sequence length was 3215 bp for all 135 isolates. Phylogenetic analysis of whole genome sequence showed that the Vietnamese HBV sequences clustered with genotypes B and C reference sequences (Fig. 1). Most HBV isolates belong to genotype B (72.6%; 98/135), subgenotype B2 (12.2%; 12/98) and B4 (87.7%; 86/98). 27.4% (37/135) of the isolates were genotype C and all genotype C isolates were belonged to subgenotype C1. The subgenotype C1 isolates formed a closely related cluster with a high bootstrapping value (99.99). There were no significant differences in genotypes among i) the isolates from 2012, 2014 and 2016, ii) isolates sequenced by Sanger or Illumina sequencing methods, iii) liver enzyme (AST and ALT) level, iv) viral load and v) geolocation of the patients. All sequences have been deposited in Gen Bank under accession numbers MF621878 and MF674382 - MF674515.

**Fig. 2:**
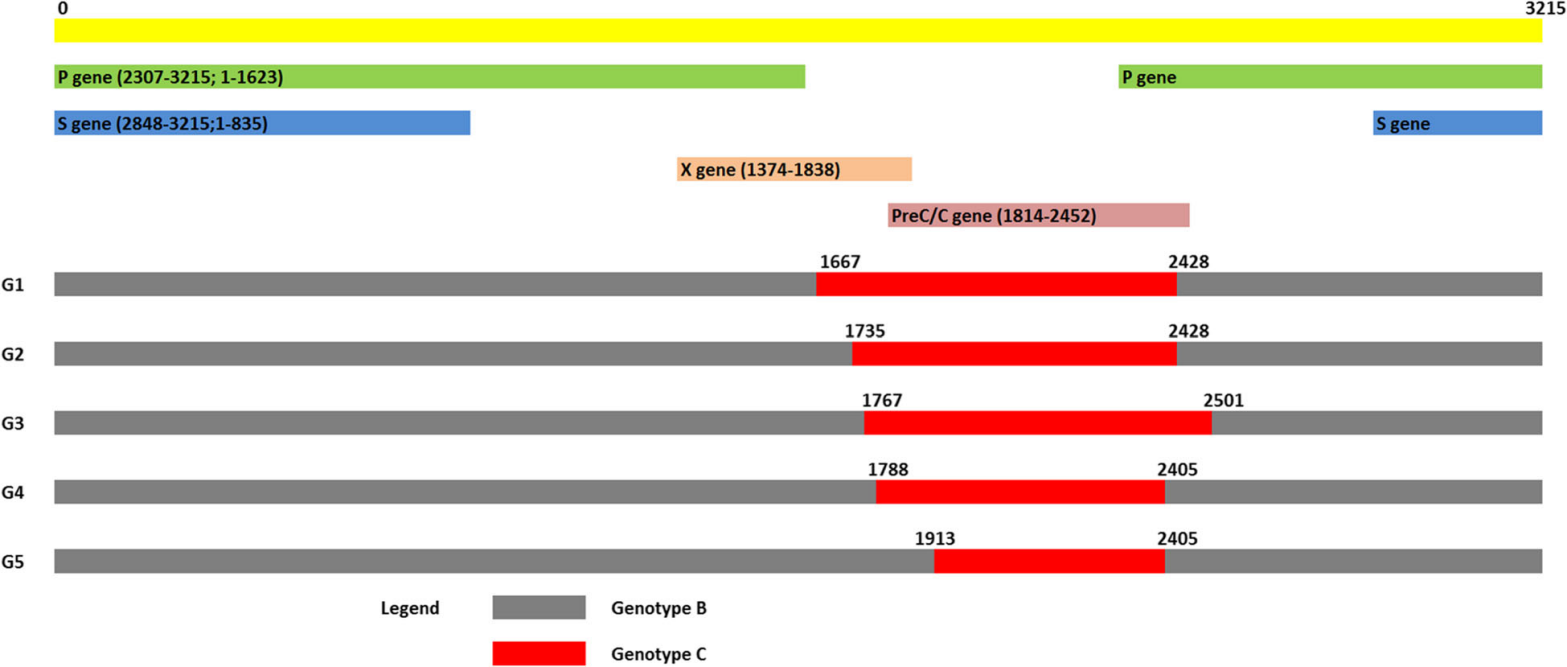
Schematic representation of the mosaic structure of the HBV recombinant sequences of the present study. The recombinant sequences are divided into five groups (G1 to G5); based on the size of the recombinant fragments. The size of the recombinant fragments were 761 bp, 693 bp, 734 bp, 617 bp, and 492 bp for Group1, 2, 3, 4 and 5 respectively. Genotype B sequence is presented in gray and genotype C sequence in red. A linear physical map of the HBV genome including the position of different gene(s) is depicted. Numbering starts from the hypothetical EcoRI restriction site

Recombination analysis using RDP identified evidence of recombination in 68.1% (92/135) of the HBV isolates including 93.8% (92/98), and 0.0% (0/37) of genotype B, C isolates respectively (*p <* 0.0001). In all recombinant isolates the minor donor was genotype C including subgenotype C1. The recombinant sequences were grouped into five groups (Group 1 to Group 5) based on the length of the recombinant fragment. Among the recombinant isolates 14.8% (20/92), 35.5% (48/92), 5.1% (7/92), 11.8% (16/92) and 4.4% (6/92) were recombinant Group 1, 2, 3, 4 and 5 respectively. The recombinant groups, recombinant fragment lengths, recombination breakpoints, and major and minor parents are shown in Fig. 2 and Additional file 2. The majority of the recombination events were identified in the X gene and in the PC/C gene.

Amino acid substitutions at primary and secondary/compensatory mutations in RT region of polymerase gene were identified in 0.7% (1/135) of the isolates. Putative mutations at NAr were observed in 16.3% (22/135) isolates including 20.4% (20/98) of the genotype B and 5.4% (2/37) of the genotype C isolates (*p* = 0.026). Besides this, we also identified genotype dependant AA polymorphisms at 7 sites. The presence of asparagine or serine at rt53, histidine or tyrosine at rt124, asparagine or aspartic acid at rt134, tyrosine or phenylalanine at rt221, valine or isoleucine at rt224, and histidine or asparagine at rt238 were significantly correlated with genotype B or C, respectively (*p* < 0.0001).Serine at 256 was significantly more associated with genotype C then B, though serine was predominant at rt256 for both genotypes.

PreS1/S2/S gene mutation: None of the isolates had *preS1* deletions or *preS1* start codon mutations. A2962G and C2964A mutations were identified in 99% (97/98) and 98% (96/98) of the genotype B isolates (Table 2). The preS2 gene start codon mutation (M1 V/T/I) was identified in 6.66% (9/135) of the isolates. N3S mutation was significantly higher in genotype C isolates (*p* < 0.001) isolates (Table 2). Among the isolates, 8.1% (11/135) had a mutation in the “a” determinant region including 2.2% (3/135) P120S/T, 2.2% (3/135) I/T126S/A, 3% (4/135) M133 L/T and 0.7% (1/135) G145R. There were no significant differences in the mutations between genotype B and C isolates (Table 2).

**Table 2:** Analysis of mutation in the preS1/S2/S gene reported to be associated with HCC and immune escape

Nucleotide	Amino acid	All isolates *N* = 135% (*n*)	Genotype B *N* = 98 v	Genotype C *N* = 37% (*n*)	*p* value
Mutation in preS1/S2 and S gene associated with HCC					
PreS1 gene					
A2962G	N38E	71.9 (97)	99.0 (97)	0.0 (0)	0.000
C2964A	N38 K	71.1 (96)	098.0 (96)	0.0 (0)	0.000
C3026A/T	A60V/E	100 (135)	100 (98)	100 (37)	
PreS2 gene					
Start codon	M1 V/T/I	6.7 (9)	8.2 (8)	2.1 (1)	0.237
T53C	F22 l	2.2 (3)	3.1 (3)	0.0 (0)	0.379
A162G	N3S	27.4 (37)	0.0 (0)	100 (37)	0.000
T531C/G	L126 T/S	77.0 (104)	98.0 (96)	21.6 (8)	0.000
S gene					
A706C	V184A	3.7 (5)	4.1 (4)	2.7 (1)	0.581
T766A	S204R	4.4 (6)	6.1 (6)	0.0 (0)	0.140
Mutation in S gene associated with immune escape					
S gene					
	P120S/E	2.2 (3)	3.1 (3)	0.0 (0)	0.379
	I/T126A/N/I/S	2.2 (3)	3.1 (3)	0.0 (0)	0.379
	M133 L/T	3.0 (4)	4.1 (4)	0.0 (0)	0.273
	G145R	0.7 (1)	0.0 (0)	2.7 (1)	0.274

BCP/preC/core gene mutation: BCP mutations at T1753C, G1757A, A1762T, G1764 T and C1766G were analyzed and 30.4% (41/135) of all isolates including 78.4% (29/37) of the C1 isolates had at least one mutation. T1753C, G1757A, A1762T, G1764A and C1766G mutations were identified in 11.1% (5/135), 7.4% (10/137), 25.9%% (35/135), 24.4% (33.135) and 2.2% (3/135) of the isolates and A1762T and G1764 T mutations were significantly more frequent in the genotype C1 isolates (*p* < 0.001) (Table 3). A double mutation (A1762T and G1764A) was identified in 67.5% (25/37) of the C1 subgenotype isolates compared to 8.2% (8/98) of the genotype B isolates (*p* < 0.001). A G1896A mutation in PC gene was present in 22.2% (30/135) of the isolates, including 30.6% (30/98) of the genotype B isolates compared to 0% of the C1 isolates (*p* < 0.000).

**Table 3:** Analysis of mutation in the BCP, precore/core gene reported to be associated with HCC in 135 HBV isolates from southern Vietnam

Nucleotide	All isolates *N* = 135% (*n*)	Genotype B *N* = 98% (*n*)	Genotype C *N* = 37% (*n*)	*p* value
Mutation in BCP region				
C1653T	0.7 (1)	1.1 (1)	0.0 (0)	0.726
T1753C	11.1 (15)	1.1 (1)	37.8 (14)	0.000
G1757A	7.4 (10)	0 (0)	27.0 (10)	0.000
A1762T	25.9 (35)	10.2 (10)	76.6 (25)	0.000
G1764A	24.4 (33)	8.2 (8)	76.6 (25)	0.000
A1762T/G1764A	24.4 (33)	8.2 (8)	76.6 (25)	0.000
C1766G	2.2 (3)	2.0 (2)	2.7 (1)	0.621
T1768A	1.4 (2)	1.1 (1)	2.7 (1)	0.475
Mutation in PC/Core				
G1896A	22.2 (30)	30.6 (30)	0 (0)	0.000
G1899A	0.7 (1)	1.1 (1)	0 (0)	0.726
A2159G	3.7 (5)	3.1 (3)	5.4 (2)	0.419
A2189C	6.7 (9)	6.1 (6)	8.1 (3)	0.469
G2203A/T	0.7 (1)	1.0 (1)	0.0 (0)	0.726

## Discussion

Determination of genotype and subgenotype of HBV is important as they are associated with clinical presentation, transmission, response to therapy and treatment outcome [2]. Considering the large pool of treatment naive chronic HBV patients and limited availability genotyping facilities in Vietnam, genotype and subgenotype data representative of the population are important for clinical decision making, including empirical therapy, disease modeling, and health resource allocation for the management of chronic HBV patients [13]. However, one of the key criteria for assignment of an isolate to a particular subgenotype should be based on analyzing the WGS. In our study we have analyzed the WGS of isolates for genotype and subgenotype determination. We used a 7.5% diversity across WGS criteria to define an isolate as a particular genotype and 4.5% to 7.5% intra-genotypic nucleotide divergence for assigning a sequence to a subgenotype [5]. We have collected 2% and 8% of the samples of treatment naïve chronic HBV patients attending to a tertiary care and hepatitis referral hospital for southern Vietnam during 2012, 2014, and 2016 in order ensure the representativeness of our data. Besides this, the geolocation analysis indicates that patients enrolled in our study were from 26 provinces including 87 districts of southern Vietnam.

Our data indicate that in southern Vietnam HBV genotype B is dominant, followed by Genotype C. This is in agreement with earlier data published from Vietnam and southeast Asia using a partial S gene sequencing approach [15]. Although HBV genotype and subgenotype have distinct geographical distributions, we could not identify such distributions in our study population. This might be due to the fact that both genotype B and C is prevalent in southern Vietnam and the subgenotype diversity is limited (i.e. only three subgenotypes are circulating in the southern Vietnam).

Recombination analysis revealed that two thirds of the isolates, including 90% of the genotype B isolates, are a genotype B/C recombinant. This is not surprising as it has been reported that HBV genotype B/Ba (B2-B5) isolates from Vietnam, China, Hong Kong, Indonesia, and Thailand have undergone recombination with HBV/C in the core promoter/precore/core genomic region [22]. It is also interesting to note that the HBV genotype B1 isolates (also called Bj) from Japan are non recombinant and are less virulent compared to HBV genotype B2-B5 (also called Ba) isolates from Vietnam, China, Hong Kong, Indonesia and Thailand [23].

We selected patients who were treatment naïve to define the presence of preexisting drug resistance mutations, and to understand the prevalence of virulence associated mutations in the population that are not influenced by treatment selection pressure. With the wide use of NAs, potential NAr mutation positions in RT region have been reported. We found that less than 1% of the HBV isolates had preexisting primary drug resistance mutations. We have identified NAr resistant mutations in 16.4% of the isolates, which are similar to earlier data from the region where 25% of the isolates had such mutations [24]. In this study seven genotype-dependent AA polymorphic positions were identified for B- and C- genotypes. This is in agreement with earlier reports from China; however we could not identify genotype-dependent AA polymorphic positions at the rt91 position. This might be due to evolution and adaption of HBV isolates in the Vietnamese population.

None of the isolates in our study had a *preS* deletion which is associated with higher risk for HCC. However, longitudinal studies showed that the *preS* deletion mutations occur during the long course of liver diseases, but not at the beginning of HBV infection [7]. As patients selected in our study are treatment naïve, it may be that they have been infected only for a relatively short time. It is interesting to note that 8.1% of the isolates had mutations in the “a” determinant region. These mutations can affect the antigenicity of HBsAg and have shown to be responsible for: i) false-negative results by commercial assays for HBsAg; ii) evasion of anti-HBV immunoglobulin therapy, and iii) evasion of vaccine induced immunity. These “vaccine-escape” mutants are more common in countries with high rates of endemic infections and universal immunization programs [25]. Further studies on the association between mutations in the “a” determinant region and vaccination status of the patients in Vietnam would provide insight in the role of these mutations.

HBV genotype C is known to be more virulent and associated with poor clinical outcome often leading to HCC. Genotype C isolates in our study belonged to subgenotype C1 and were tightly clustered with high prevalence of known virulence mutations (e.g. A1762T & G1764 T double mutation) [15]. Approximately one quarter of the genotype B patients had a G1896A mutation. This mutation creates a premature stop codon in the *PC* gene, prevents translation of PC protein, and abolishes the production of HBeAg and results in HBeAg seroconversion. This is in agreement with earlier reports indicating a low HBeAg positivity and earlier HBeAg seroconversion in genotype B isolates [26].

Data on transmission of HBV are lacking in Vietnam. However, perinatal/vertical transmission is reportedly more common in genotype B and C compared to A, D and E-J [26]. Taken together our data suggest that along with the predominant HBV subgenotype B4, subgenotype C1 variant is also circulating in southern Vietnam and of public health significance.

A limitation of our study was the lack of clinical data on the patients. Such data would support the clinical relevance of the genotype and subgenotype. Longitudinal studies on patients with specific genotype and subgenotype infection are essential to fill this knowledge gap.

## Conclusions

HBV genotype B and C predominates in Vietnam. Among genotype B and C isolates, B4 and C1 subgenotype are predominating respectively. Subgenotype C1 isolates contains significantly more virulence associated mutations compared to genotype B isolates.

## Additional files

Additional file 1: Geolocation mapping of the 135 chronic HBV patients enrolled in the study. Description: Addresses of each enrolled patient were mapped by QGIS v2.18 at ward level. Geolocation of wards of genotype B patients are marked red, genotype C patients as blue and wards with patients of both genotype B and C are marked as green (DOCX 319 kb)

Additional file 2: Recombination analysis. Recombination analysis of the HBV isolates from this study using RDP4 v 4.85 program. Recombination analysis of the 92 HBV isolates from this study using RDP4 v 4.85 program. An isolate was considered recombinant if detected by 5 out of 6 program (RDP, BootScan, Max Chi, Chimaera, SisScan and Topol). Recombinant isolate, minor parents and identity, recombinant break points, size of the recombinant fragment and location of the recombination are presented. (PPTX 141 kb)

Additional file 3: HBV reference isolates_180817. The HBV reference genome sequence and sequences isolated from Vietnam in this study used for phylogentic analysis. Gene Bank accession number and subgenotype of the reference sequences used for phylogenetic analysis. Gene Bank accession number and subgenotype of the HBV isolates from this study. (DOCX 12 kb)

## Abbreviations

AA: Amino acid
ALT: Alanine aminotransferase
AST: Aspartate aminotransferase
BCP: Basal core promoter
DNA: Deoxyribonucleic acid
dNTPs: Deoxynucleoside triphosphates (dNTPs)
HBV: Hepatitis B virus
HCC: Hepatocellular carcinoma
HTD: Hospital for Tropical Diseases
IU: International Unit
LC: Liver cirrhosis
NA: Nucleos(t)ide analogue
NAr: Nucleos(t)ide analogue resistance
ORFs: Open reading frames
PC: Pre core
PCR: Polymerase chain reaction
RT: Reverse transcriptase
SNP: Single nucleotide polymorphism
UNL: Upper Normal Limits
WGS: Whole genome sequencing
