# Supplementary material for: Molecular characterization of hepatitis B virus in Vietnam
Source: BMC Infect Dis. 2017 Aug 31;17:601. doi: 10.1186/s12879-017-2697-x (PMC5580302; doi:10.1186/s12879-017-2697-x)
Supplement: Supplementary file 1 — Geolocation mapping of the 135 chronic HBV patients enrolled in the study. Description: Addresses of each enrolled patient were mapped by QGIS v2.18 at ward level. Geolocation of wards of genotype B patients are marked red, genotype C patients as blue and wards with patients of both genotype B and C are marked as green (DOCX 319 kb) [file 12879_2017_2697_MOESM1_ESM.docx]

Supplementary file 1


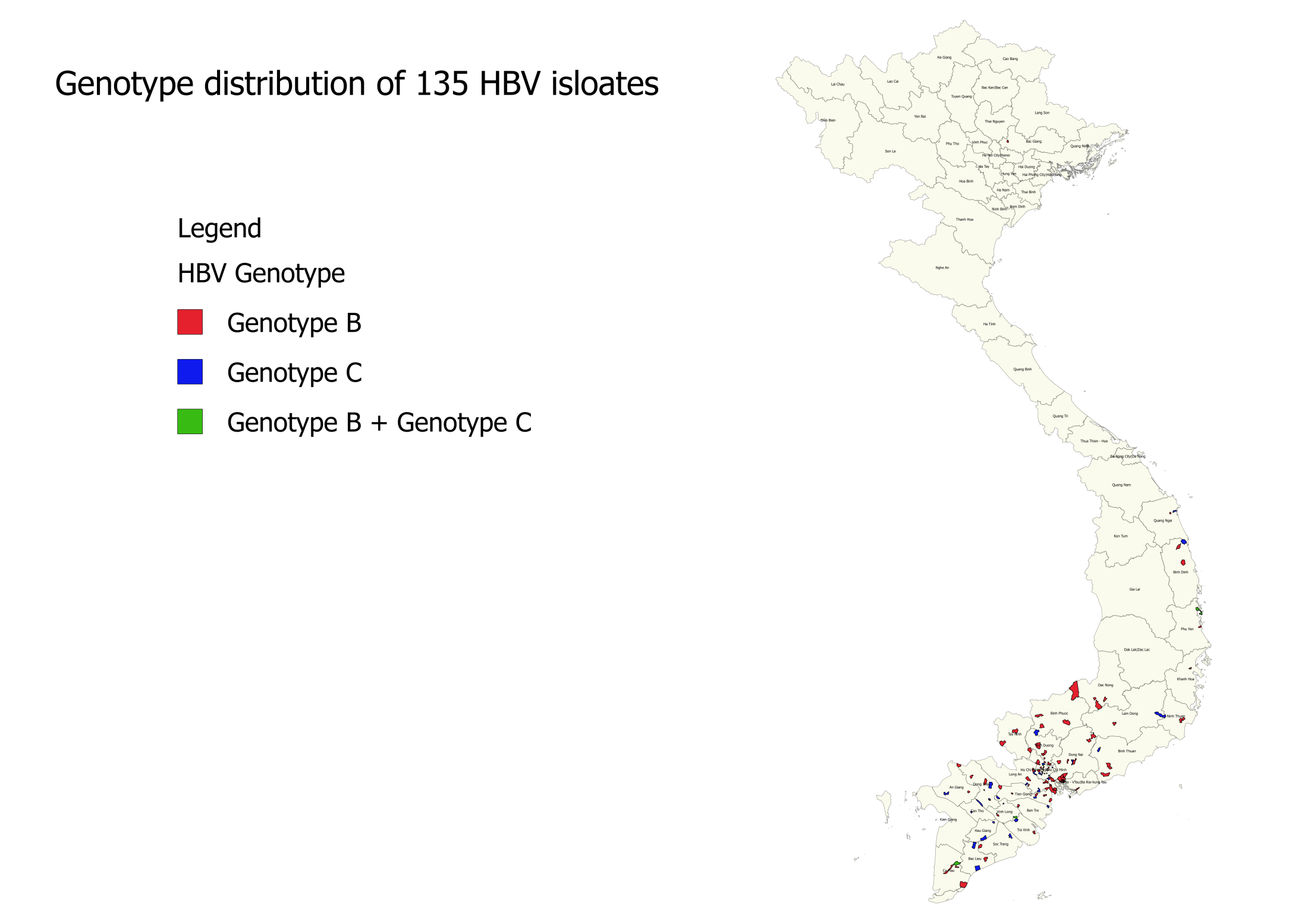


Supplementary file 1: Geolocation mapping of the 135 chronic HBV patients enrolled in the study. Addresses of each enrolled patient were mapped by QGIS v2.18 at ward level. Geolocation of wards of genotype B patients are marked red, genotype C patients as blue and wards with patients of both genotype B and C are marked as green.
