# Supplementary figures and images for: Molecular characterization of hepatitis B virus in Vietnam
Source: BMC Infect Dis. 2017 Aug 31;17:601. doi: 10.1186/s12879-017-2697-x (PMC5580302; doi:10.1186/s12879-017-2697-x)

## Slide 1
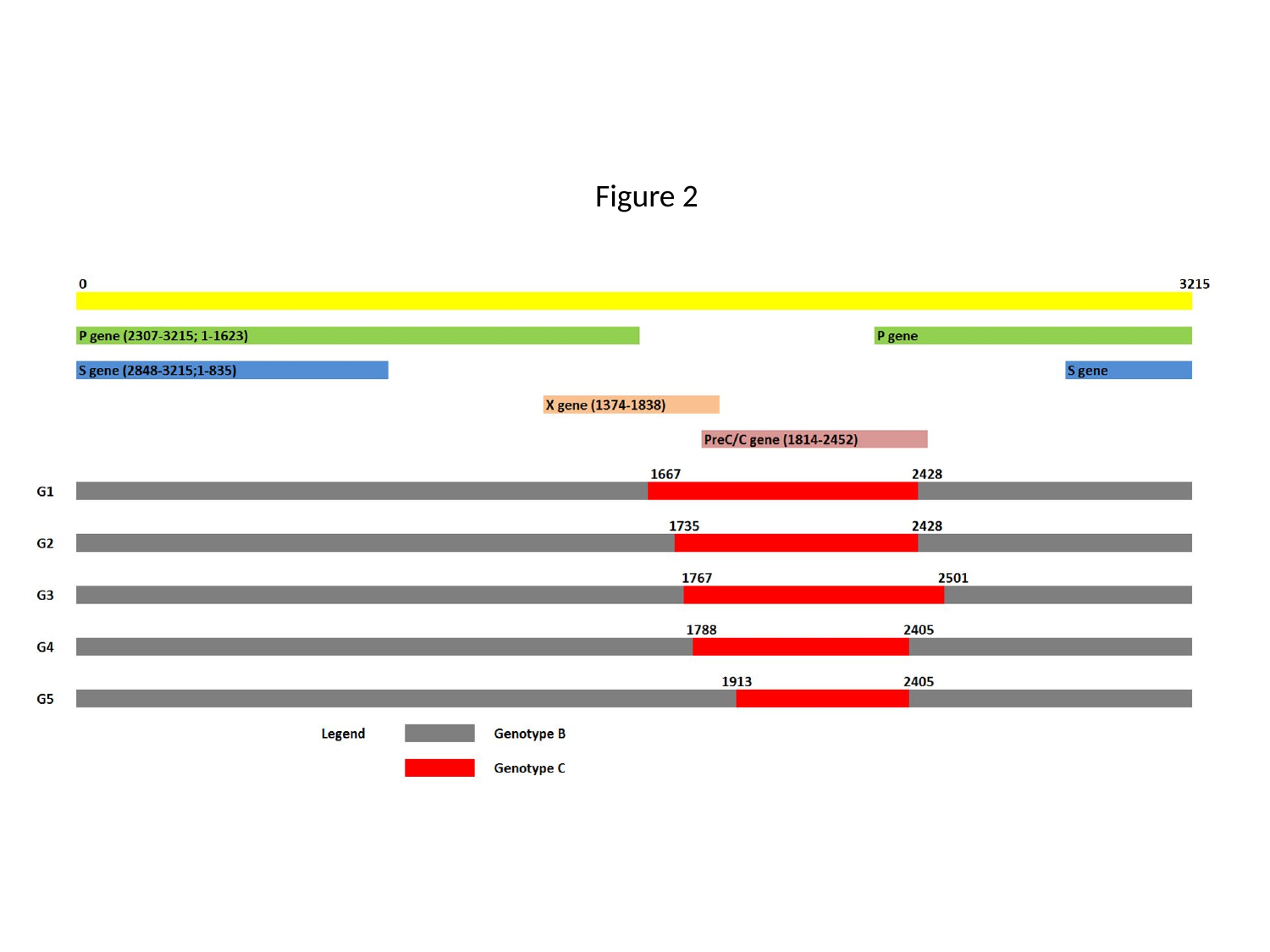

Figure 2

Supplement: Supplementary file 2 — Recombination analysis. Recombination analysis of the HBV isolates from this study using RDP4 v 4.85 program. Recombination analysis of the 92 HBV isolates from this study using RDP4 v 4.85 program. An isolate was considered recombinant if detected by 5 out of 6 program (RDP, BootScan, Max Chi, Chimaera, SisScan and Topol). Recombinant isolate, minor parents and identity, recombinant break points, size of the recombinant fragment and location of the recombination are presented. (PPTX 141 kb) [file 12879_2017_2697_MOESM2_ESM.pptx]
