# Supplementary material for: Molecular characterization of hepatitis B virus in Vietnam
Source: BMC Infect Dis. 2017 Aug 31;17:601. doi: 10.1186/s12879-017-2697-x (PMC5580302; doi:10.1186/s12879-017-2697-x)
Supplement: Supplementary file 3 — HBV reference isolates_180817. The HBV reference genome sequence and sequences isolated from Vietnam in this study used for phylogentic analysis. Gene Bank accession number and subgenotype of the reference sequences used for phylogenetic analysis. Gene Bank accession number and subgenotype of the HBV isolates from this study. (DOCX 12 kb) [file 12879_2017_2697_MOESM3_ESM.docx]

Supplementary file 3

The HBV reference genome sequence accession number and subgenotype used for phylogentic analysis: JN182318: A1; HE576989: A2; AB194951: A3; AY934764: A4; FJ692613: A5; GQ331047: A6; FN545833: A7; AB642091: B1; FJ899779: B2; GQ924617: B3; GQ924626: B4; GQ924640: B5; JN792893: B6; GQ358137: B7; GQ358147: B8; GQ358149: B9; AB697490: C1; GQ358158: C2; DQ089801: C3; HM011493: C4; EU410080: C5; EU670263: C6; AB483838: C6; AB483838: C6; AB483841: C6; AB483842: C6; AB483843: C6; AB483844: C6; AB483847: C6; GU721029: C7; AP011106: C8; AP011104: C8; AP011105: C8; AP011107: C8; AP011108: C9; AB540583: C10; AB554019: C11; AB554020: C11; AB560661: C11; AB560662; AB554025: C12; AB554018: C12; AB644280: C13; AB644281: C13; AB644284: C14; AB644286: C15; AB644287: C16; GU456636: D1; GQ477452: D2; EU594434: D3; GQ922003: D4; GQ205377: D5; KF170740: D6; FJ904442: D7; FN594770: D8; JN664942: D9; FN594748: E; FJ709464: F1b; DQ899146: F2b; AY090459: F1a; DQ899142: F2a; AB036920: F3; AF223965: F4; GU563556: G; AB516393: H; FJ023659: I1; FJ023664: I2 ; AB486012: J and AY226578: Woolly monkey as an out-group.

Isolate identification number and subgenotype of 135 HBV isolates from the present study:

HBV040003 : B4; HBV040005: B4; HBV040008: B4; HBV040009: B4; HBV040010: C1; HBV040013: B4; HBV040015: C1; HBV040016: B4; HBV040019: C1; HBV040022: B2; HBV040023: B4; HBV040024: B4; HBV040025: B4; HBV040026 : B4;HBV040027: B4; HBV040028: B4; HBV040029: C1; HBV040030: C1; HBV040031 : B4; HBV040033: B4; HBV040034: C1; HBV040035: C1; HBV040037: B4; HBV040038: B4; HBV040042: B4; HBV040043: B4; HBV040044: C1; HBV040047: B4; HBV040048: B4; HBV040049: C1; HBV040052: C1; HBV040058: B4; HBV040060: B4; HBV040061: B4; HBV040063: B4; HBV040065: C1; HBV040067: C1; HBV040069: B4; HBV040071: B4; HBV040075: B4; HBV040076: B2; HBV040078: B4; HBV040080: B4; HBV040081: C1; HBV040083: B2; HBV040084: B2; HBV040085: C1; HBV040086: C1; HBV040087: C1; HBV040090: B4; HBV040093: B4; HBV040099: B4; HBV040100: B4; HBV050003: C1; HBV050007: B4; HBV050008: B4; HBV050023: B4; HBV050024: B4; HBV050025: B4; HBV050034: B4; HBV050050: C1; HBV050051: B4; HBV050052: C1; HBV050055: B4; HBV050056: B4; HBV050057: B4; HBV050059: B4; HBV050060: B2; HBV050067: B2; HBV050068: B4; HBV050071: B4; HBV050074: B4; HBV050076: C1; HBV050077: B4; HBV050079: B4; HBV050080: C1; HBV050081: C1; HBV050082: B4; HBV050083: B4; HBV050084: B4; HBV050085: B4; HBV050086: C1; HBV050087: B4; HBV050091:B2; HBV050092: B4; HBV050094: C1; HBVM0015: C1; HBVM0614: B4; HBVM1014: B4; HBVM1314: C1; HBVM1414: C1; HBVVZ0514: B4; HBVVZ0614: B4; VN02A0424: C1; VN02A0764: C1; VN02A0827: B4; VN04A0023: B4; VN04A0142: B4; VN04A0296: B4; VN04A0313: B2; VN04A0315: B4; VN04A0321:B4; VN04A0352: B4; VN04A0380: C1; VN04A0438: B2; VN04A0451: C1; VN04A0864: B4; VN05A0034: B4; VN05A0145: C1; VN05A0515: B4; VN05A0949: B4; VN05T0114: B4; VN05T0131: B4; VN05T0132: C1; VN05T0139: B4; VN05T0140: B4; VN05T0141: B4; VN05T0156: B4; VN05T0158: B4; VN05T0168: B4; VN05T0189: C1; VN05T0190: C1; VN05T0191: B2; VN05T0200: C1; VN05T0203: B4; VN05T0217: B2; VN05T0218: B4; VN05T0223: B4; VN05T0226: B4; VN05T0230 : B4; VN05T0240: B4; VN05T0250: B2; VN05T0253: B4; VN05T0254: C1; VN05T0261: B4

The accession number for the sequences in gene bank is MF621878 and MF674382 - MF674515
